# The Challenges of a Complex and Innovative Telehealth Project: A Qualitative Evaluation of the Eastern Quebec Telepathology Network

**DOI:** 10.15171/ijhpm.2017.106

**Published:** 2017-09-13

**Authors:** Hassane Alami, Jean-Paul Fortin, Marie-Pierre Gagnon, Hugo Pollender, Bernard Têtu, France Tanguay

**Affiliations:** ^1^Institute of Health and Social Services in Primary Care, Research Center on Healthcare and Services in Primary Care of Laval University (CERSSPL-UL), CIUSSS-Capitale Nationale, Quebec City, QC, Canada.; ^2^University Hospital Center of Quebec-Laval University Research Center, Quebec City, QC, Canada.; ^3^Faculty of Medicine, Laval University, Quebec City, QC, Canada.; ^4^Faculty of Nursing, Laval University, Quebec City, QC, Canada.; ^5^Integrated Health and Social Services Centre of Chaudière-Appalaches Hôtel-Dieu de Lévis, Lévis City, QC, Canada.

**Keywords:** Telepathology Network, Telehealth Implementation, Evaluation, Sustainability, Healthcare Services

## Abstract

**Background:** The Eastern Quebec Telepathology Network (EQTN) has been implemented in the province of Quebec (Canada) to support pathology and surgery practices in hospitals that are lack of pathologists, especially in rural and remote areas. This network includes 22 hospitals and serves a population of 1.7 million inhabitants spread over a vast territory. An evaluation of this network was conducted in order to identify and analyze the factors and issues associated with its implementation and deployment, as well as those related to its sustainability and expansion.

**Methods:** Qualitative evaluative research based on a case study using: (1) historical analysis of the project documentation (newsletters, minutes of meetings, articles, ministerial documents, etc); (2) participation in meetings of the committee in charge of telehealth programs and the project; and (3) interviews, focus groups, and discussions with different stakeholders, including decision-makers, clinical and administrative project managers, clinicians (pathologists and surgeons), and technologists. Data from all these sources were cross-checked and synthesized through an integrative and interpretative process.

**Results:** The evaluation revealed numerous socio-political, regulatory, organizational, governance, clinical, professional, economic, legal and technological challenges related to the emergence and implementation of the project. In addition to technical considerations, the development of this network was associated with major changes and transformations of production procedures, delivery and organization of services, clinical practices, working methods, and clinicaladministrative processes and cultures (professional/organizational).

**Conclusion:** The EQTN reflects the complex, structuring, and innovative projects that organizations and health systems are required to implement today. Future works should be more sensitive to the complexity associated with the emergence of telehealth networks and no longer reduce them to technological considerations.

## Background

Telehealth has the potential to improve access to care and the continuity of health services, especially for rural and remote areas, commonly called “medical deserts.”^1^ Because of its geographic and demographic situation and the drive of some leaders, the eastern part of the province of Quebec (Canada) has been historically innovative in the field of telehealth. This is the context in which the Eastern Quebec Telepathology Network (EQTN), one of the largest in the world in terms of the number of participating organizations and jurisdictions,^2,3^ emerged.

Telepathology is a specialized field of telehealth. In short, telepathology is “*the electronic transmission of pathological images, usually derived from microscopes, from one location to another, for the purpose of interpretation and diagnosis*.”^4^ It involves the practice of anatomopathology (commonly called “pathology”) at a distance. Telepathology can be used to establish a histopathological diagnosis, obtain a second medical opinion, or provide distance training by means of *information and communications technologies* (*ICTs*).^4-7^ Telepathology allows a number of specialists (eg, pathologists and surgeons) and organizations (service recipients/requester and service providers/respondents) to network, by facilitating an exchange of clinical-administrative data and images in digital format, and sharing expertise. Telepathology can help improve access, quality, continuity, and efficiency of pathology services, particularly in rural and remote areas.^8-13^ However, telepathology, like any innovative and structuring telehealth project, is accompanied by major changes and transformations inherent to the growing importance of ICTs in health systems reform and reorganization strategies.

This article describes the results of the EQTN evaluation and aims to identify and understand factors that have influenced the implementation, operation and results of this complex and innovative project. We present an analysis of the socio-political, regulatory, organizational, governance, clinical, professional, economic, legal and technological dimensions that conditioned and oriented its planning, emergence, development, and implementation, as well as the conditions for its success, sustainability, deployment and expansion.

### The Quebec Health System

In Canada, the organization and management of health policies are under provincial jurisdiction. The Federal Government contributes to the funding of provincial health systems through federal transfers that are conditioned by the respect of certain conditions.^14^ In Quebec, the health system is close to the Beveridgian model: mainly tax-funded, public, universal and almost free from the point of view of service users.^15^

With respect to governance, two main levels are present: (1) the Ministry of Health and Social Services (MHSS), which ensures close coordination and regulation by setting priorities, objectives and allocation of resources; and (2) the Institutions that deliver services. They include 22 Integrated Health and Social Services Centers (IHSSC) that result from a larger merging process to integrate, on a territorial basis, all the different functions and institutions related to health and social services, including hospitals and primary care services. Nine of them also include a university mission related to research, teaching and evaluation. They are designed as IUHSSS (U = university). IHSSC/IUHSSS provide the majority of public primary care and social services. The Institutions also include four university hospitals (UH) that deliver for the province specialized and subspecialized services and two specialized University Institute for Cardiology. They have a supraregional mission that requires them to cover several health regions. The four UH have always been central for the development of telehealth in Quebec. IHSSC/IUHSSS and hospitals are funded mainly in the form of overall budgets, based primarily on past expenditures, although activity-based funding is being generalized.

Quebec has also created Integrated University Health Networks (IUHN), attached to the four faculties of medicine in the province. Their role is to foster complementarity, cooperation and integration of healthcare organizations with a university mission and the universities to which they are affiliated. Their role has been associated on a territorial basis. They have been given a special active role to foster telehealth development and implementation.

Finally, there are also medical clinics and family medicine groups that primarily provide general medical services and may, in some cases, provide more specialized services. For the vast majority of their primary care or hospital services, physicians are mainly paid by activity and are bound by service agreements with the organizations where they work.

### The Eastern Quebec Telepathology Network

In 2004, the Quebec MHSS asked each of the four IUHN of the province to prioritize two telehealth projects. The IUHN-Laval University (IUHN-LU) has opted for telepathology (for more technical and clinical information about this telepathology project see Têtu et al^17^ and Perron et al^18^). This choice resulted from the fact that the supply of pathology services has become a problematic issue in Quebec due to the province’s vast size and the uneven geographical distribution of the population. This situation is explained by the following: (1) a lack of staffing and difficulties in recruiting and retaining pathologists in remote areas; (2) difficult working conditions for pathologists when it comes to ensuring continuous services, due to the lack of replacement staff during holidays and difficult travel in winter; and (3) an over-specialization in pathology which, because of the risk of under-utilization of pathologists’ cutting edge expertise, makes it difficult to practice in small communities. This situation is also impacting the recruitment and retention of surgeons and other clinicians requiring pathology services in hospitals where there are no pathologists.

According to MHSS figures, the IUHN-LU (1.7 million inhabitants for a territory of 410 000 km^2^) numbered 59 pathologists in 2017. This represents a shortfall of at least five pathologists when it comes to providing coverage of all service requests, so leading to important waiting times that can affect quality of patient care.^19^ This situation has remained relatively stable for several years. Among IUHN-LU pathologists, some 59,3% (35/59) are located in the capital (Quebec City) region.

The others (24/59) are distributed unevenly across a vast territory and their long-term retention is uncertain, especially given the age of most practicing pathologists.

**Figure 1:**
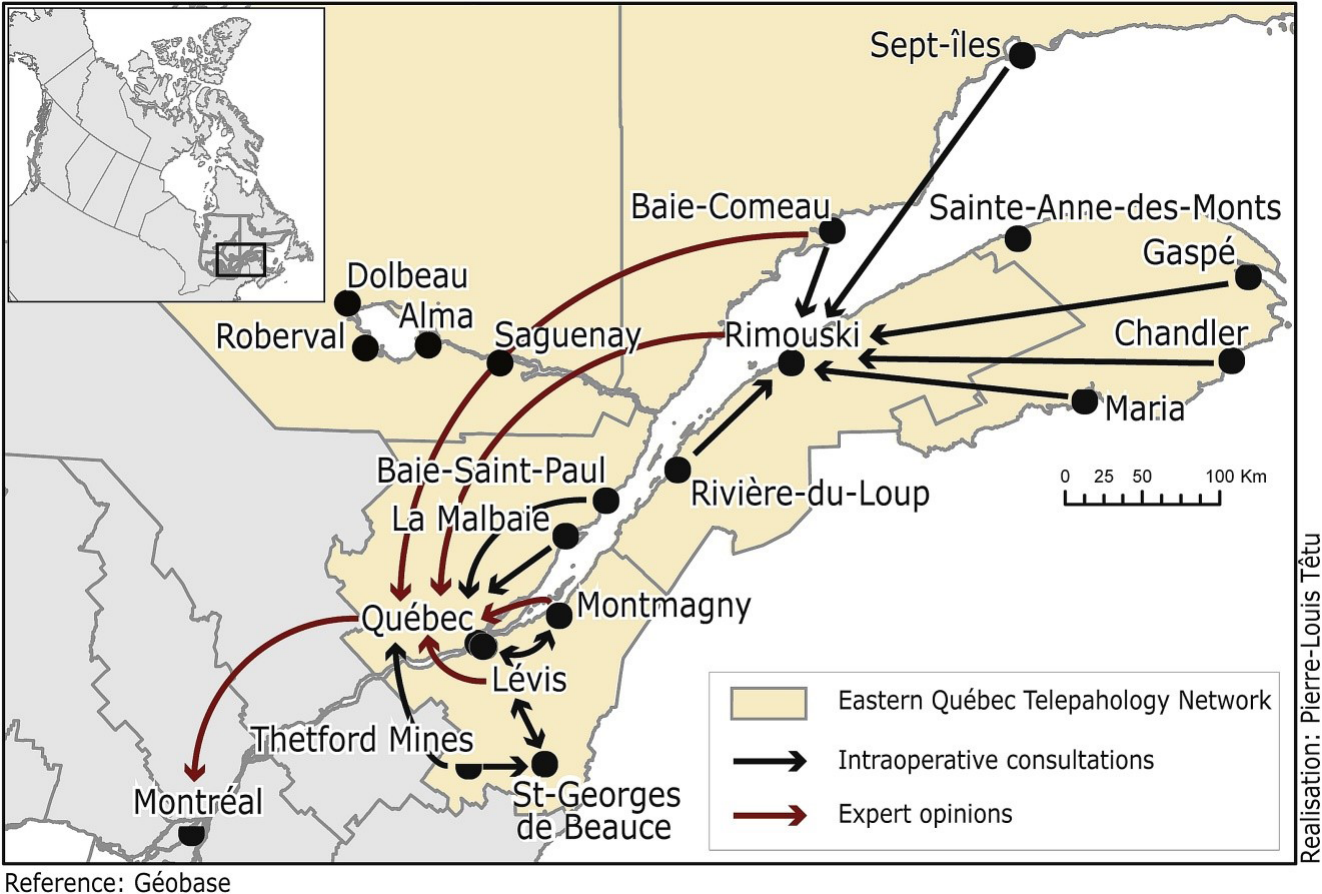
The Territory Covered by the EQTN, at a Given Time, With Examples of Interactions Between the Different Participating Organizations.^20^

Abbreviation: EQTN, Eastern Quebec Telepathology Network.

Initiated in 2006, the EQTN was jointly funded by the MHSS and Canada Health Infoway (CHI) in early 2008 with a non-recurring budget of slightly more than CA$6.2 million. This funding covered mainly technological devices. In 2011, the first clinical uses were initiated. The EQTN currently has 22 participating locations, including the University Hospital of Quebec-Laval University (UHQ-LU), which has a supraregional mission with a “safety net” role, which results in coverage of specific requests for services from other participating regions (Figure 1).

Unlike several similar projects, where the tertiary center is responsible for answering all requests for services from remote areas (eg, University Health Network Telepathology in Ontario^21^), this project has opted for a strategy based on inter-organizational and interregional collaboration. Indeed, it networks, both vertically and horizontally, organizations from the same region as well as those from different regions. The other strategy consists of targeting and being limited to services responding to urgent needs of an extemporaneous and a second opinion nature, especially in oncology. This strategy was seen as beneficial for the population and is sensitive to the issue of cancer and adapted to the payment conditions of the funders.

The EQTN project was designed to implement a clinical telepathology network as a viable and effective solution to providing support for IUHN-LU hospitals in order to avoid shortages in pathology services and to expand the range of services provided.

### Evaluation: Scope and Objectives

The specific EQTN evaluation project was initiated in 2013. It was intended to provide information for decision-making regarding project orientations and improvements to be implemented for its successful deployment within a perspective of sustainability and scaling-up across the province.

The main objectives of this evaluation were the following: (1) to study the functioning of the project in relation to the actors, issues, and strategies used; (2) to better understand the results and effects of use on access, continuity and quality of services and work, service organization, and practice transformation; (3) to explore socio-political, regulatory, organizational, governance, clinical, professional, economic, legal and technological factors influencing implementation, adoption and use, and ultimately the sustainability and dissemination of telepathology; and (4) to identify conditions that may be useful to ensure better integration and diffusion of telehealth in health systems.

## Methods

### Evaluative Approach

To take into account the characteristics of the project – innovative, complex, dynamic, and evolving – and decision-makers’ need to monitor and integrate the lessons of the evaluation into their decision-making processes, we opted for a case study, particularly appropriate when the focus of study cannot be separated from its context.^22,23^

We adopted a utilization-focused evaluation approach^24^ whose aim is the following: (1) *descriptive and explanatory*, to determine how, according to certain rules, stakeholders cope and deal with complex phenomena^25^; (2) *comprehensive*, eg, to take stock of all facts and issues related to the unfolding of the project^26,27^; (3) *participatory and pluralistic*, to include the perspectives of the various stakeholders, partners, and actors involved in the project^24,28^; (4) *progressive (developmental) and formative*, in order to ensure co-construction, field support, and translation of knowledge in action with all the actors, taking into account the different stages of the project^29^; and (5) *summative*, to assess the achievement as regards the initial objectives.

### Conceptual Framework

**Figure 2:**
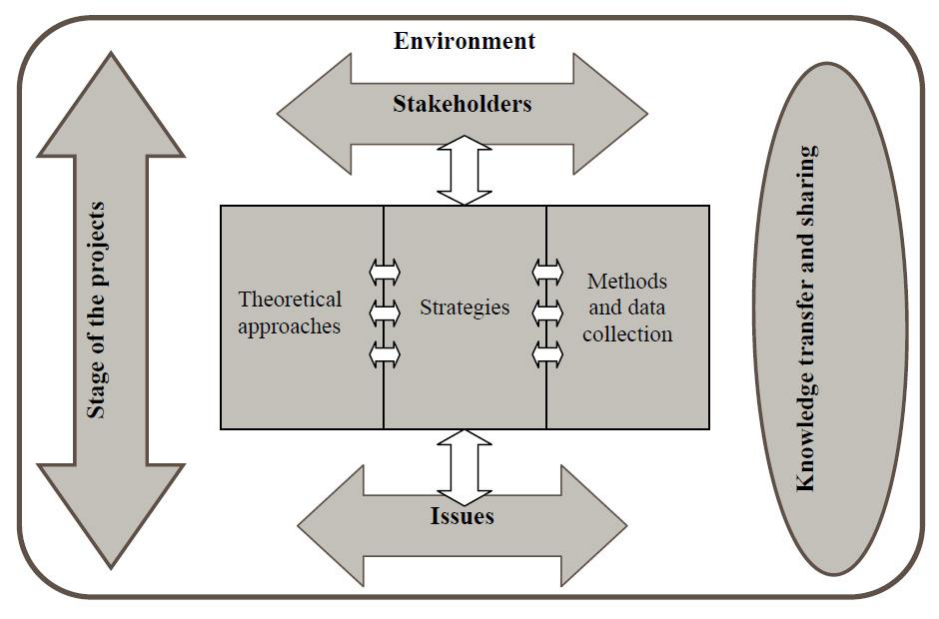
Strategic Framework for a Useful and Used Evaluation.^30^

To structure the approach, ensure its integration into the overall project, and complete the proposed evaluation plan, we used the *Strategic framework for a useful and used evaluation* (Figure 2).^30^ This framework makes it possible to take into account the characteristics and different stages of the project, the actors and stakeholders, the environment, the issues, as well as the different levels of intervention. It also facilitates the choice of methods and the evaluation and knowledge-sharing strategies to be adopted as appropriate given the innovative nature of the project.

### Data Collection Activities

Data used for the evaluation included (1) a historical analysis, over a 10-year period, of all documentation related to the project (eg, newsletters, minutes of meetings, activity tracking and monitoring documents, protocols and guidelines, periodic presentations, use evaluation reports, articles, and documents from government and funding agencies), the goal of which was to reconstruct the sequence of key events and situate them in their context, while highlighting the critical decisions and the reasons they were made, as well as the role of the different actors in the evolution of the project; (2) participant observation of meetings of the governance committee in charge of the project; and (3) interviews, meetings, discussions, and exchanges (formal and informal) with project stakeholders, including policy-makers, managers, clinical, technological, and administrative project officials, pathologists, surgeons, and technologists.

The people interviewed were identified primarily by the project team as well as by way of project documents and reports. Internet searches were also conducted to identify other key people in participating organizations. Participant selection was guided by the need to gather a diversity of perspectives from the different stakeholders involved in the project.

Interviews were recorded and transcribed verbatim. Subsequently, verbatim, documents and observation notes were subjected to a qualitative thematic content analysis,^31^ using a deductive-inductive approach based on the framework and new themes emerging from the data, with the help of NVivo-10 software.^32^ Analyses were carried out by HA and HP; a third person was able to intervene when there was a difference in perspective in the case of divergence (JPF). In order to increase our interpretation and analysis capacity, we cross-checked the results from the different sources described above, in line with the principle of data triangulation.^33^ Thus, we were able to formulate and re-evaluate our conclusions by regularly returning to the primary sources of data to detect possible variations and convergences/divergences.^31,34^ This approach made it possible to verify, qualify, and complete our findings and observations. Moreover, the proximity to the project of certain authors also made it possible to establish trust reports, which allowed for communication and contact with all actors. This element also forced us to adopt a reflexive approach throughout the evaluation, in particular by having a critical look at all the data collected.^35^

Given our evaluation mandate within a healthcare organization, we have applied the principles of the “*Guidelines for Ethical Conduct*” recommended by the Canadian Evaluation Society (integrity/honesty, accountability, confidentiality, respect, and responsibility for people’s welfare).^36^

## Results

We conducted 19 interviews (9 clinicians, 7 managers and decision-makers, and 3 technologists) and made 3 observation site visits. We observed and participated to 4 project team meetings (2 hours each), 2 biannual meetings involving participating locations (3 hours each), and 15 meetings of the telehealth executive committee – including the MHSS representatives – where the telepathology project was on the agenda (2.5 hours each). We also had access to the project documentation (project planning, project operations manual, articles related to the project, use evaluation report, minutes of meetings, use tracking reports, project newsletter, telepathology clinical guidelines, international telepathology cooperation documents, pathology and surgery medical staffing plans, closing report of the project and ministry’s telehealth governance plan).

The same information was often derived from interviews, meetings, documentation, or observation. In fact, we applied triangulation principles and merged these data into a coherent or integrative-interpretative synthesis. The results are structured according to the following criteria (from the evaluation framework): (1) use and effects; (2) clinical, professional and human aspects; (3) organizational aspects; (4) governance and strategic aspects; (5) technological aspects; (6) legal aspects; and (7) economic and financing aspects.

### Use and Effects

Important evaluations were made by colleagues, mainly focused on the planned uses of telepathology, were conducted during the project.^3,20,37,38^

Our study added more information. Indeed, for the expected effects, data confirm that two-stage surgeries, transfers of patients from remote areas to urban centers, as well as service breaks in critical situations, were avoided. Improvements in medical care and diagnostic delay were also mentioned. Telepathology also helped curtail pathologist travel to several locations, a phenomenon which has resulted in clinical time gains.

Our evaluation showed that several teams used the technology for applications that were not initially planned, including for emergency biopsies, macroscopies, routine histologies, immuhistochemistry, cytology, education, and even teleautopsy. According to surgeons, telepathology could also be relevant for other clinical activities and other specialties (eg, endoscopy, gynecology, and orthopedics).

*“(...) In endoscopy, we have never used it, while there are certain polyps that would benefit from being analyzed on site by telepathology. These are pieces that become damaged during back and forth travel and so become difficult to analyze (...)”* (R1) [all interview quotes have been translated from French to English].

Also, the use of technology has been influenced by a combination of factors, including the evolution of scientific evidence and changes in clinical protocols, which have had a significant impact on the scope of the project:

*“(...) Surgeons’ requests have changed. The most important demands were for sentinel lymph nodes (for breast cancer). (...) Following certain knowledge advances and according to [new] protocol, a lymph node is not important if the tumor is smaller than one centimeter. This phenomenon has greatly reduced the demand for telepathology (...). A resulting decrease in activity in this regard has been in the order of 27% (...)”* (R2).

### Clinical, Professional, and Human Aspects

Interpersonal relationships have been central in the decision as to whether or not to use telepathology. Indeed, much of the activity has emerged through existing trust relationships involving surgeons/pathologists/technologists. These include people from the same organization, as well as from two or more different organizations.

In addition, thanks to the dynamic described above, new practices procedures were developed and negotiated locally between pathologists and surgeons from different organizations (requester and respondent) to facilitate work involving telepathology beyond existing clinical protocols. These alliances and partnerships have evolved over time and vary according to the context.

*“(…) We [pathologist-ENT, and surgeon] have established standards and criteria that help everyone (surgeon/pathologist/technologist). For example, it’s the surgeon who sets the tone in the operating-room (...)”* (R3).

Surprisingly, telepathology was found to be of little help when it comes to promoting the recruitment and retention of pathologists in remote areas. It has also been reported that pathologists increasingly want to practice as a team. This issue concerns the quality of the work environment. On the other hand, telepathology has enabled recruiting and retaining surgeons in certain locations. Some surgeons mentioned that they would not have joined the organization or would have left it without telepathology.

Additionally, team dynamics were important, and even decisive, for the use (or non-use) of telepathology in networked work contexts or more centralized (or concentrated) organization contexts. For example, in one of the most active locations of the project, new pathologists could be trained in the use of telepathology by local team members in favour of the approach.

*“(...) All the pathologists in the location in question (...) use telepathology. This allows everyone to acquire experience in this regard; they can exchange information about the cases they come across via telepathology. They collaborate extensively, thus enhancing their expertise. Everyone can develop their expertise (...)”* (R2).

This example illustrates the extensive support of some clinicians who worked on the project and enabled the necessary collaboration initiatives and partnerships for the development of telepathology services. Indeed, it appears that the decision to use telepathology is not only based on organizational will. Because of their professional autonomy, pathologists and surgeons decide to use (or not to use) telepathology and do so, to a greater or lesser extent, regardless, or not, of the organizational decision.

Moreover, the dynamic collaboration of the “pathologist-technologist-surgeon” trio has been highlighted as decisive in the success or failure of telepathology. The evolution of this trio and the transformation of roles and responsibilities were underlined in the context of virtual network. In this sense, the importance of technologists in the telepathology services chain has been highlighted. It has also been reported that the role of technologists (eg, nature and load of work, responsibilities) was not sufficiently taken into account at the beginning of the project. In fact, when it comes to telepathology, technologists are required to digitize glass slides, take certain samples, and carry out procedures usually reserved for pathologists.

This situation was observed in several locations in remote areas that do not have pathologists. In such locations, technologists have become the “extension of the pathologist’s arm” in the operating room (OR), given that no pathologist is actually on hand. In these locations, technologists have come to work closely with the surgeon, who in the past worked with a pathologist physically present in or close to the OR. Thus, the use of telepathology has relied strongly on the skills of technologists. According to some interviewed clinicians, it is important to improve the expertise of technologists so that they can carry out activities requested by the remotely located pathologist. For the latter, this competence also creates trust within the pathologist/technologist/surgeon trio.

This new working configuration where the technologist has a preponderant role raises other professional (eg, reserved acts), economic (eg, expertise recognition and remuneration), and legal (eg, responsibility) questions and issues. For example, we observed that in one of the participating rural hospitals without onsite pathologist, a technologist found herself working as a “Laboratory Head.” With the use of telepathology, she carried out activities that were usually reserved for the pathologist. This technologist had a crucial role when it came to maintaining a certain level of surgical activity in this hospital.

This last point raises the issue of the recognition of a “new” status for technologists working in remote hospitals that do not have pathologists but that do offer surgical services. As such, the question of training and accreditation has been highlighted. As part of this project, the positive dynamic in some environments has led some pathologists to (informally) train technologists to use telepathology. Technologists have seen their work evolve and have to some extent become *“pathologists’ assistants.”* However, this status is not yet recognized in Quebec.

We have also observed the emergence of new modes of practice. One pathologist brings along digitized glass slides to read during his frequent travels between two provinces. Technology has also allowed pathologists who are unable to travel (eg, for family reasons or because of inclement weather) to work from home.

The project has also faced certain difficulties in ensuring coverage of second-opinion or sub-specialized services, particularly in academic hospitals. Pathologists felt they were overwhelmed by the usual demands for services within their own organizations. Their fear of being overwhelmed by even more requests for services from other organizations partly explains their reluctance to use telepathology. However, other pathologists believe that telepathology could improve practice conditions in these centers, thus ensuring greater supraregional availability for these services.

Moreover, we observed that most of the clinician and manager “champions” who were able to implement and give legitimacy to the project are near retirement. Thus, the question of availability and training as regards the next generation of clinicians and managers is critical, and some interviewees pointed out the vulnerability of such an “individual-dependent” project. The risk of loss of “organizational memory” and “experiential wisdom” related to the project is significant. It was also reported in meetings and interviews that many professionals, including surgeons and dermatologists, were unaware of the project, or did not see its relevance or usefulness, when in fact they could have used it in some clinical situations. Promotion of the project and communication about it thus appear to have been insufficient.

### Organizational Aspects

The project was designed to help improve the organization of services, not only for pathology but also for surgery. However, it was reported from various data sources that the link between telepathology and the organization of IUHN-LU pathology and surgery services was unclear. The role of pathology, telepathology, and even telehealth on a broader scale in service organization plans and strategic orientations was murkier still.

The project was also highly dependent on changes in medical staff, especially the extensive mobility of pathologists in regional and sub-specialized hospitals. This mobility has impacted the supply and organization of services, especially since the critical mass of clinicians in the various settings was very low. This situation has created significant differences in the ability of hospitals to plan and provide in-house services or respond to requests from other organizations. Staff movement has been observed when it comes to telepathology itself, where it has contributed to a trend toward concentration of pathologists in a “regional hub.” This mobility has resulted in readjustments and changes in existing alliances, agreements, and collaborations among certain organizations.

*“(...) Telepathology was an encouraging and catalytic element in my leaving the (...) [former organization]. For me, by joining (...) [a new organization], there was the possibility of covering all the regions through this regional hub where telepathology would have a role to play (...)”* (R2).

At the same time, organizations in the regions have expressed fears of losing their physicians following the implementation of telepathology and that pathologist positions would likely be transferred to regional centers where services could be provided via telepathology. The fact that at least two organizations which were using telepathology indeed lost their pathologist added credibility to this fear, regardless of the real reason of their departure. Organizations have thus expressed the need to be better informed on the management of pathologist positions in light of telepathology. This point was not actually taken into account when the project was implemented.

At another level, issues have been raised in relation to requests for services. For example, the question of OR management has been reported in documents, meetings and interviews. A frozen section session via telepathology may take more time than a session with the pathologist onsite (eg, to digitize glass slides, different communication mode). According to current performance and efficiency criteria, this situation may not allow for optimal OR management, either for the organization or for the surgeons whose remuneration does not take this element into account. This situation partly explains the fact that hospitals continue to use “itinerant” pathologists for frozen section sessions scheduled for a particular day of the week. However, this performance criterion has been deplored by other clinicians and managers. The latter point out that even if a frozen section session lasts one hour, this procedure is always more efficient than transferring the patient or performing a second surgery (eg, a surgeon may first operate on a patient to obtain a specimen. He sends it to the pathologist for analysis. The answer may take several days or weeks. The patient will then be operated a second time when the analyses are available.). Furthermore, the decision of discontinuing the use of telepathology for frozen sections should not be taken before solutions to improve the efficiency have been investigated. However, no such improvement procedure has been planned with the implementation.

It was also found that organizing collaborative ventures that involved requesting and respondent locations varied according to the expertise, interests, and needs of the various organizations. The main point here is that the diversity of local contexts has led teams and organizations to work differently.

### Governance and Strategic Aspects

The evaluation highlighted several elements that go far beyond the project under review. As for telehealth in general in Quebec, the planning and macro-management of the project were centralized and mostly “techno-centered,” in particular concerning the MHSS and some regional boards. Some clinicians explained their refusal to use telepathology given this “top down” approach where they were not consulted, nor sufficiently integrated into the project:

*“(…) Physicians refused telepathology because it was imposed from the top [the Ministry and the organization] and they were not consulted beforehand. It should not be forgotten that doctors have autonomy with respect to their practice (...)”* (R4).

Furthermore, the nature and definition of the roles, responsibilities, type and degree of involvement, and influences of the various participant organizations were unclear. For example, the supraregional responsibility of the academic hospital of reference was not clear to all the players, in particular concerning how it should fulfill its “safety net” role in order to ensure coverage for subspecialty services in remote areas.

In this case, the nature of incentives and conditions for service coverage were unclear for all organizations. Based on our information, no real procedure has been put in place to prioritize cases to be analyzed among organizations and their pathologists. In fact, service priorities are still primarily oriented toward in-house cases, where the pathologist works, instead of more urgent cases in another hospital. This last point puts into question the true nature of the contracts between organizations participating in the network, and therefore the obligation to comply with them.

On another level, there was a consensus that the approach of those providing funding was relatively narrow with respect to project operation and technology use. The administrative constraints inherent to the performance indicators required for payment by sponsors were regularly brought up. The project team considered these indicators to be poorly adapted to local realities and opportunities and felt that they did not take the unique nature of each context into account. This situation has created difficulties for teams that have tried to adapt and align technology to their context, as well as to the evolution of their local needs and expertise. Managers and clinicians reported that a more open approach to the use of telepathology would have favored a more successful adoption and use of available technology:

*“(...) The calculation is biased; the information received is used for calculation. These are real applications, but there are a lot of applications, such as support to macroscopic, that are not counted and don’t appear anywhere in the statistics. And when you look at the official figures, it gives the impression that it’s stabilized or that it doesn’t progress (...)” (*R5).

In addition, many questions related to service contracts (eg, interoperability and archiving) have been raised. Managers and clinicians strongly agreed on the importance of setting up a national registry for the management, storage, and archiving of virtual slides. This national registry would lead to harmonize practices and better coordinate and integrate services.

In the same vein, it was also reported that a catalog of telepathology services should be developed to enable organizations to learn about all the services available. Managers and clinicians also found it essential to identify the various possible interconnections between this project and other provincial digital health initiatives, notably the “Quebec Health Record,” whose objective is to facilitate the collection, conservation, and consultation of patient-related medico-administrative  information.

### Technological Aspects

The speed of the high performance digitizer would not allow for more than one slide to be scanned every three minutes, which could slow pathologists’ ability to respond to requests made through telepathology. The digitising speed is however largely dependent on whether slides are being scanned at 20x or 40x and some pathologists prefer the highest power despite the additional time required. According to our observations and through our discussions and meetings with clinicians and project teams, serious questions were also raised regarding file size, speed of transmission, and archive locations. In addition, it was pointed out the need to have dynamic and sufficiently flexible systems to be able to take account the evolution of clinical and organizational needs.

Another major barrier highlighted was that the technological systems of the different organizations and those used by clinicians in their homes are not interoperable:

*“(...) For example, when it comes to hospital (Y) and the other hospitals in the region, each has its own operating system for pathology. They are not able to communicate with each other at all. (...) We also have a pathologist from hospital (X) who helps us by working from home, but she cannot connect because she doesn’t have the same system (…)”* (R4).

### Legal Aspects

The project’s clinical director raised a number of forensic issues concerning the use of telepathology. He has asserted that without the clarification of certain issues, the adoption and use of telepathology could be difficult.

In Quebec, the legislator requires the creation of a record both by the service requester and the service provider. This is also the position of the Canadian Medical Protective Association (CMPA). However, this requirement differs greatly from the current nature of the practice where pathologists receive e-mail requests. In this case, the receiving pathology department proceeds only with the creation of a request in the computer system, but does not create or open a record. In addition, the Act respecting health services and social services (ARHSS-2005) and the College of Physicians of Quebec require that two organizations that collaborate and use telehealth must sign an agreement. This situation is also new in pathology because traditionally pathologists who receive requests for consultation by regular mail do not require that an agreement is signed between the organizations involved.

In addition, the CMPA recommends that all images examined in telepathology be stored in accordance with current conservation schedules. For organizations, this recommendation would imply to have substantial space to store these images. The Clinical Director also questioned the necessity and relevance of conserving all the images produced by telepathology. However, it pointed out that virtual images likely to be the subject of litigation could be fully preserved.

### Economic and Financing Aspects

It was out of the scope of the present evaluation to quantify the systemic added value of telepathology. However, we have identified different elements that seem to be necessary for a future medico-economic evaluation of this project, such as situations where patient transfers and two-stage surgeries have been avoided. In addition, cases where the technology has been used for services that were not planned initially (eg, teleautopsy) appear to have added value. However, the absence of monitoring to report these activities poses a challenge to conduct such evaluation.

The performance criteria and real gains for the requesting organization are unclear. Indeed, it is the responding organization that accounts activity, and therefore receives the funding associated with it. This situation could lead some organizations to refuse to use pathologists’ services via telepathology. In addition, it was stressed the importance of looking at the impact on the cost of patient management for the organizations involved.

*“(...) It’s nice to not transfer the patient, but then we are stuck with him in our hospital (...) [to keep a patient involves costs for the organization] (...)”* (R6).

On this point, it was also observed that some participating organizations wanted to offer telepathology services to increase their volume of activity, which would ultimately allow them to have other pathologist positions and increased budget. Thus, the redistribution of savings achieved through telepathology between organizations poses several challenges (eg, nature of incentives, performance criteria for the requesting and the responding sites).

With respect to remuneration, it was found that establishing a telepathology network should require reviewing current payment modes, both for physicians (pathologists and surgeons) and technologists (valuation of activity). Indeed, telepathology implies other ways of functioning, such as teleworking for some pathologists. For surgeons, due to technological (eg, to digitize glass slide) and cognitive reasons that affect communication, telepathology takes somewhat longer time than when the pathologist is physically present in the OR (for its part, the Perron et al,^18^ study reported that the average time difference is not great, but still superior). This is particularly true when slides are being scanned at 40x whereas the additional time required is much less when slides are scanned at 20x. This situation implies a somewhat longer use of the OR, which raises questions of financial compensation for overtime, but also questions of OR management for the organization with the economic impact that it implies.

Finally, according to interviews, meetings and documents, limited funding does not consider the need to finance new equipment and new service modalities that are necessary for the sustainability and scaling-up of this network. This raises once again the question of the availability of recurrent funding. This element does not appear to have been taken into account at the emergence of the project, as it was initiated in response to an opportunity to fund telehealth projects from a federal funding organization (CHI). The fact that the project’s funding was of a limited and non-recurring duration, in addition to being mainly technology-oriented and not service-oriented, raises concerns for the follow-up of the project. Therefore, if it stops, the network would be threatened, at least in its present form.

## Discussion

The project evaluated is unique by its nature, objectives and expectations. Because of its complex, innovative and structuring character, this project has contributed to addressing the concerns that are not normally addressed in initiatives that aim to provide efficient, effective, continuous and quality telehealth and health services for populations.

Telehealth involves major adjustments of organizational, professional, clinical and technological issues to be processed so to provide adequate services within an integrated and coordinated health system. Diverse and varied dimensions make technology - as a technical object - “secondary” in the midst of a complex health ecosystem, with often blurred contours. Thus, healthcare organizations are one of the most complex forms of social systems.^39,40^ In addition, the health system is a type of “professional bureaucracy” characterized by great decentralization and professional autonomy.^41,42^ New technology is thus introduced into social, organizational, and cultural environments with their individual histories and routines. For technology to be properly integrated, these systems must reorganize and restructure to discover a new equilibrium that would allow them to continue to evolve over time. Telehealth is a striking example of this phenomenon because it directly affects the provision of health services, which are at the core of the health system.

### A Complex and Structuring Project

The project has attempted to network 22 locations, each with its own specific characteristics and dynamics: geographical (rural vs. urban), organizational, administrative, professional, and technological. This situation creates challenges of considerable magnitude when it comes to harmonizing and aligning various systems and processes, especially in an integrated service network vision.

This project shows also that the practice of pathology is inseparable from the practices of other clinicians, especially surgeons. Thus, a transformation of clinical practices and organization of services are required in order to be part of a comprehensive vision of the health system, where the alignment between specialties, modes of practice, and professional and organizational cultures, without forgetting local and regional contexts, is central to harmonizing and integrating services using technology as a lever. Harmonizing the strategic plans of participating organizations with MHSS strategies, policies, and priorities is also required, a further response to the “vagaries” of political contexts and government changes. For example, as we finalized the evaluation, Quebec underwent a major health system centralization reform that has disrupted the telepathology network and participant organizations. Indeed, with this reform, there was another project for the centralization of medical biology laboratories, which certain telepathology stakeholders were not aware of, although this project included the closure of a number of laboratories. This is the typical example of the disconnection and not alignment between different priorities, strategies and levels of governance.

This project cannot moreover be separated from its spatial and temporal context. Indeed, the project has evolved in light of new knowledge acquired on the ground and from other international experiences along the way, and it has been affected by regulatory and technological factors. As such, the project requires capacities, means, and leeway for action and reaction to deal with unpredictable events and developments at the clinical, organizational, political, and technological levels.

On the other hand, the project is also characterized by negotiations and exchanges involving a variety of actors and stakeholders who may have divergent visions and objectives and who maintain their autonomy and flexibility in an environment where all the actors remain ultimately interdependent.

### Project Strengths

### Clinical, Organizational and Technological Leadership

Undoubtedly, the strength of this project lies in its strong leadership at various levels (clinical, organizational, and technological). Indeed, responsibility for the project has remained in the hands of a team with extensive experience in the field and a history of close collaboration with the players involved. This helped establish a climate of mutual trust, making it possible to address the various issues related to the progress and direction of the project in a serene climate, despite the many challenges and issues to be overcome over the course of the project.

One of the noteworthy elements to highlight is the fundamental role of the clinical dimension at all levels and stages of the project. Indeed, strong clinical leadership helped overcome many of the challenges encountered during the project, notably those stemming from the initial vision as driven by the funders, a vision that was relatively rigid and mainly focused on specific uses of technology. The actors in the field have rethought and redefined this vision to anchor it in a clinical and organizational approach, going beyond the simple implementation of technology.

Moreover, strong clinical and organizational leadership has fostered a favorable dynamic with respect to the use of technology. On the other hand, we also found that this same leadership could be mobilized against the project itself. In fact, some clinicians were responsible for the refusal of certain organizations to use telepathology.

### Collaboration and Innovation

Furthermore, the project created dynamics of collaboration and mutual assistance between clinicians and organizations. As a result, we have seen the construction of clinical platforms (refers to a model of clinical collaboration) adapted to local realities and organizational needs. This flexibility and dynamism in the field have led to the emergence of other uses for the technology. This was in part possible because the project favored a strategy capitalizing on local and regional collaborations and dynamics where the academic hospital acted as a “safety net” and not as the main distributor of services as is often the case for telepathology services.

The bottom-up expertise was validated and recognized, thanks to a vitality of local teams, which can be considered as innovation laboratories that integrate clinical, organizational, and technical dimensions. Local teams were able to innovate and experiment using an exploratory approach more oriented toward their needs, going beyond what was initially planned in the project (eg, use of technology to do teleautopsy or teleformation). This process has made it possible to develop other ways to work and collaborate, using technology. Consequently, new roles and responsibilities have been negotiated locally to capitalize on the potential provided by telepathology. Such local dynamism can be seen as one of the conditions for project success. However, given all these local innovations, it is very difficult to implement and operationalize a single service model for all participating locations.

### Challenges and Conditions for Sustainability

### Challenges Inherent to the Organizational Transformations

Telepathology, and digital pathology, is accompanied by changes in structures, the organization of services, and clinical processes. It calls into question pre-existing organizational and professional cultures. In fact, it brings with it other forms of communication, which involve a reconfiguration of relations between clinicians, technologists, managers, and indirectly, patients, as well as between all these actors and the organization itself.

Telepathology, at least in the Quebec context, requires technologists to develop significant expertise that allows them to become a central link in the proper functioning of the telepathology context in locations where no pathologists are on hand. This new situation raises issues regarding task delegation (eg, activities normally reserved for pathologists), and therefore their eventual validation. Technologists now perform what is called a “critical function,” which involves an interruption, even a paralysis, of the organization’s workflow if not performed.^42,43^ This said, the sustainability of telepathology is partly dependent on whether the organizations and other professional actors are able to accept, recognize, and formalize this new role of technologists.

On the other hand, telepathology involves a repositioning of relations and interactions between the actors themselves (eg, pathologists, technologists and surgeons), as well as between actors and the organization (eg, pathologists and their organizations). The novelty here is that the use of technology forces organizations to go beyond their physical dimension to integrate a wider environment, thus forming a sort of network-organization which is more structured around information flows and more and more “virtual teams.”

Nevertheless, the viability of these new networks depends on the nature and clarity of the cooperation routines that will be set up among the various actors (professional and organizational) concerned. Indeed, the question of confidence in change seemed decisive for such a restructuring to be possible and sustainable. In this instance, negotiation, in the logic of change management, takes on a central role in order to build a new configuration of relationships and interactions, which in turn leads actors collectively to learn, innovate and adopt new modes of practice, communication and work.^43^ It is also important for organizations to develop a learning culture and become sufficiently flexible so as to benefit from the experience on the ground.

### Technology as “Use”

Telepathology also has an interactive character, which means that the professionals concerned, through their use of the technology, participate in its definition (or redefinition) by accepting it in its initial form, transforming and adapting it to their contexts and needs, or simply rejecting it.^44^ Moreover, this innovative mode of practice is introduced in an environment where it confronts existing modes and habits of practice and organization. As such, we are left with socio-technical systems where the technology interacts with a socio-cultural environment, in particular the organization.^45,46^ Thus, it should be kept in mind that the technology could be subject to change due to its association and confrontation with other ideas and realities specific to each organizational and practice context.^47^ Recipients can design and use the technology in a different way than originally intended because each can interact with innovation in a way that differs from others, even if they belong to the same profession.^47,48^ In other words, it is use that gives value to the technology.^49-51^

In this project, the emergence of new local clinical platforms was an adapted response to organizational and regional needs and realities, which may differ according to context and influenced by systemic issues. It was also a way for local teams to reach the objectives of covering the services expected by the MHSS. These local clinical platforms have enabled the emergence and consolidation of an inter-organizational and inter-regional dynamic that can be seen as a foundation for the development of an integrated, functional, and efficient national telepathology network.

### The Challenge of Scale-up and Sustainability

The biggest challenge for the EQTN is to transition to the required scale and become sustainable. The first consideration remains the concrete support of decision-making authorities with a telehealth and health system vision and strategy, which is not entirely the case because of the lack of a global telehealth strategy in Quebec. This support should be materialized, in the short term, through the training and availability of innovation-sensitive human resources and to be galvanized by the clinical and organizational leadership required to enable an understanding of the complexity of health system transformations in the light of telehealth. This condition is essential in making available the support required for the rapid development of applications and uses of technology.

The prospect of sustainability also implies that several elements have to be deepened and many constraints overcome. This includes the need to find a new balance in the services provision model, which is conditioned by the extent to which stakeholders in the health system are able to agree on new dynamics in the production and delivery of services. This said, the standard top-down approaches limit creativity and the latitude circles needed to innovate and to uncover new practices.

Other issues with respect to ensuring EQTN sustainability were raised during this evaluation. First, it is important to consider the conditions for taking into account local needs and the local context on a broader scale. Institutionalization should not reduce the field’s capacity to adjust and adapt. Second, a central factor for EQTN sustainability remains the availability of recurrent funding. Indeed, one of the problems regularly encountered in telehealth projects is that they are initiated with provisional funds, where sustainability is not taken into account. In other words, the project funding will eventually become an end in itself for the initiators and not a means for improving the provision of services.^52^ Funding strategies should foster longer-term approaches with sufficient flexibility to innovate (eg, avoid budgets aligned with so-called closed indicators). Computerization in health involves complex and slow transformations that take place over the long term, an approach that current funding models generally do not enable.^52^ Finally, the conditions of practice and remuneration of pathologists, surgeons, and technologists whose practices and responsibilities are impacted by telepathology must be better defined.

## Conclusion

This work, which evaluated a telepathology network’s emergence, implementation and operation, helps to inform decision-makers on the importance of taking into account the complexity stemming from changes in models of production, delivery, and organization of health services with telehealth. The implementation of integrated telehealth networks requires a clear understanding of the professional, organizational, and political dynamics and synergies that exist between all stakeholders (individual and organizational) involved. Having a systemic vision that takes into consideration the complexity of health systems is also crucial. Future research-evaluation must therefore be more sensitive to the complexity associated with the emergence of telehealth networks and no longer reduce them to solely technological considerations. Developmental-accompanying evaluation should be recognized as a lever to improve understanding of how these networks evolve in the dynamic, ever-changing environments that health systems represent and, ultimately, contributing to decision-making process.

## Acknowledgments

This work is part of an evaluation mandate received by Dr. Jean-Paul Fortin’s team. It was funded by the University Hospital Center of Quebec-Laval University, Quebec City, QC, Canada. It was also supported by scholarships (H. Alami) from: (1) the Research Center on Healthcare and Services in Primary Care of Laval University; (2) the “Conseil Franco-Québécois de Cooperation Universitaire (CFQCU)”/ the “Fonds de recherche du Québec-Nature et technologies (FRQNT)”; and (3) the Strategic Training Fellow in Transdisciplinary Research on Public Health Interventions “Promotion, Prevention and Public Policy (4P)” of the Canadian Institutes of Health Research and of the Quebec Population Health Research Network.

The authors would like to thank the “The Eastern Quebec Telepathology Network” authority and team for their availability and contribution throughout this work. Their willingness to share their experience and expertise to improve practices and advance telehealth is an important element to highlight.

Special thanks to all people (researchers, decision-makers, clinicians, managers, technologists, etc) and institutions that have contributed to the realization of this work. We would also like to thank the reviewers for their valuable comments and suggestions.

## Ethical issues

We have applied the principles of the “Guidelines for Ethical Conduct” recommended by the Canadian Evaluation Society (integrity/honesty, accountability, confidentiality, respect, and responsibility for people’s welfare).

## Competing interests

Authors declare that they have no competing interests.

## Authors’ contributions

JPF, HA, and MPG conceived and designed the evaluation plan. JPF, HA, and HP were responsible for data collection. JPF, HA, HP, MPG, BT, and FT have been involved in data analysis and interpretation of results. HA, JPF, MPG, HP, BT, and FT were engaged in the drafting of this manuscript and they all read and approved the final manuscript.

## Authors’ affiliations

^1^Institute of Health and Social Services in Primary Care, Research Center on Healthcare and Services in Primary Care of Laval University (CERSSPL-UL), CIUSSS-Capitale Nationale, Quebec City, QC, Canada. ^2^University Hospital Center of Quebec-Laval University Research Center, Quebec City, QC, Canada. ^3^Faculty of Medicine, Laval University, Quebec City, QC, Canada. ^4^Faculty of Nursing, Laval University, Quebec City, QC, Canada. ^5^Integrated Health and Social Services Centre of Chaudière-Appalaches Hôtel-Dieu de Lévis, Lévis City, QC, Canada.

## Key messages

**Implications for policy makers**

- Telehealth is primarily a health system transformation challenge: the importance of a vision and strategy to improve the organization, coordination, financing and integration of health care and services using technology as a lever.
- Political will, organizational, clinical, administrative and technological leadership are central to ongoing innovation: complementarity and synergy between the stakeholders and the different health system governance levels in a co-construction and co-evolution approaches adapted to project various stages and sustainability.
- Telehealth involves changes in cultures, models of production and delivery of services, communication modes, practices and uses: the importance of managing change in a collaborative, participatory and inclusive approach of all actors (political, professional and organizational).
- Technology should be thought from an open-ended perspective: needs and uses are what gives value to the technology (eg, end users may find and adapt other applications not originally foreseen).
- Developmental-accompanying evaluation is essential to better understand the complexity and dynamics of IT projects. It makes it possible to share knowledge and support decision-making throughout the life of the project and beyond.

**Implications for the public**

This article allows the reader to have a global vision and overall understanding of the inherent issues of a complex telehealth network with a systemic dimension. Indeed, it sheds light on certain conditions that must be met and challenges that need to be addressed so that populations and the healthcare system can benefit from the potential of telehealth in improving access, continuity and quality of health care and services.
